# Eosinophilic Dermatosis of Hematologic Malignancy

**DOI:** 10.7759/cureus.65842

**Published:** 2024-07-31

**Authors:** Gabriella Beharry, Rob Lawless, Muna Shakhashiro, Kristopher McKay, Chase L Wilson

**Affiliations:** 1 Dermatology, University of Kentucky College of Medicine, Lexington, USA; 2 Internal Medicine, University of Kentucky College of Medicine, Lexington, USA; 3 Pathology, Skin Path Solutions, Atlanta, USA; 4 Dermatology, Elkhorn Dermatology, Georgetown, USA

**Keywords:** dermatological manifestation of malignancy, dermatologic manifestations, hematologic malignancies, medical dermatology, eosinophilic dermatosis, eosinophilic dermatosis of hematological malignancy

## Abstract

Eosinophilic dermatosis of hematologic malignancy (EDHM) is a rare chronic skin condition commonly affecting individuals with underlying hematologic malignancies, most notably chronic lymphocytic leukemia (CLL). EDHM presents as pruritic, insect-like bites, but without patient-reported contact/bites of insects. We present a case of a 44-year-old male who presented to Elkhorn Dermatology with a scaly rash and serpiginous borders on the nasal tip and right cutaneous upper lip. The patient was diagnosed with CLL one year prior and had been on zanubrutinib for 10 days since presenting to the dermatology clinic. Initial treatment with antifungal and antibiotic therapies showed no improvement, leading to a punch biopsy that revealed perivascular and periadnexal lymphocytic dermatitis with eosinophils. This finding, along with the patient's underlying CLL, led to a diagnosis of EDHM. This case highlights the diagnostic challenges and therapeutic complexities associated with EDHM in patients with hematologic malignancies.

## Introduction

Eosinophilic dermatosis of hematological malignancy (EDHM) is a rare dermatological condition affecting both men and women and is characterized by the presence of tissue eosinophilia in the setting of hematological malignancies [1]. EDHM is typically described as a non-specific dermatosis with pruritic erythematous papules, vesicles, or bullae that mimic insect bites on the body [2]. The non-specific nature of this dermatosis makes it difficult to diagnose because it can appear as an insect bite, or the dermatitis can mimic the cutaneous findings seen in malignant and premalignant blood dyscrasias, including leukemia, myelodysplasia and myeloproliferative disorders [3]. It is suggested that EDHM is most likely due to a dysregulated immune system driving a T-cell and eosinophil-mediated hypersensitivity reaction [3,4]. A study conducted by Almeida et al. evaluated nine cases of patients diagnosed with EDHM and found that leukemic cells act as bystander cells in the dermis and cause immune dysregulation via a process of tropism by disrupting adhesion molecules [1]. Treatment options are limited but consist of antihistamines, chemotherapy, dapsone, interferon alpha, intravenous immunoglobulin, phototherapy, and even doxycycline [2,4]. We present a case of EDHM in a male patient mimicking an insect bite reaction located on the nasal tip.

## Case presentation

**Figure 1:**
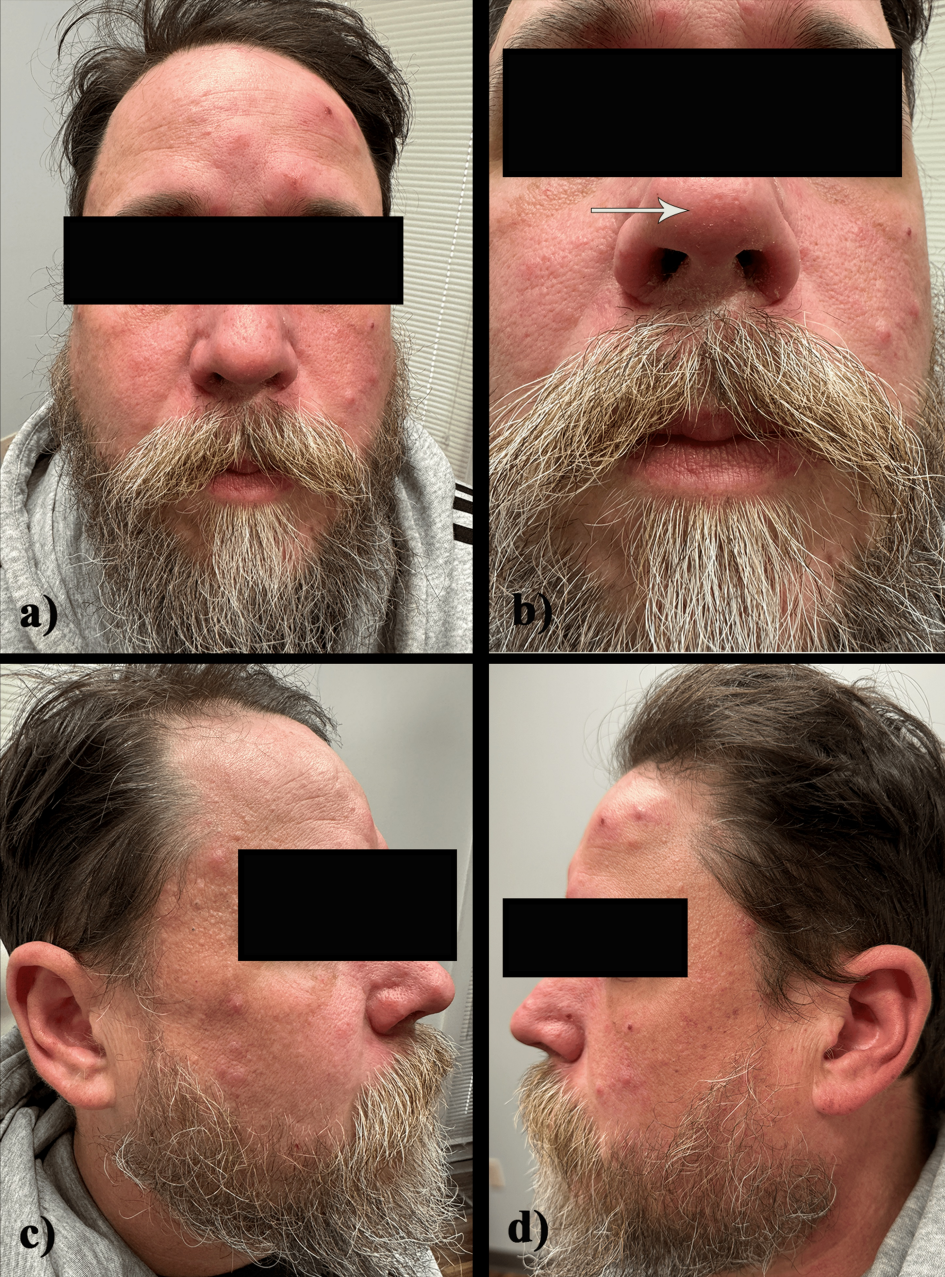
(a) and (b) Scaly patches with a serpiginous border located on the nasal tip. (c) and (d) Erythematous papules and nodules on the bilateral cheeks and temples.

A 44-year-old male presented to Elkhorn Dermatology with a rash located on the nasal tip and right cutaneous upper lip that he reports has been present for one year. On examination, the rash presented as scaly patches with a serpiginous border located on the nasal tip and right cutaneous upper lip (Figure 1), with pink folliculocentric papules and pustules located on the right naris. One year ago, the patient was diagnosed with CLL and has recently started on Zanubrutinib for 10 days before presenting to the clinic in September. It is unclear whether the rash and CLL diagnosis had a similar onset, but the rash appeared one year ago, and the patient was diagnosed with CLL one year ago as well.

During the initial visit, tinea facei and impetigo remained high on the differential, so the patient was started on terbinafine 250 mg daily for 14 days, during which his symptoms did not change significantly. Following his 14-day course of terbinafine 250 mg daily, the patient was switched to doxycycline 100 mg twice daily for 14 days. At the initial visit, a bacterial culture was also performed on the nasal tip, which returned positive for MSSA infection. Therefore, the differential diagnoses at the time favored bacterial folliculitis, tinea facei, and impetigo, but drug reactions, CLL-related opportunistic infections, and inflammatory dermatosis still remained in our differential.

**Figure 2:**
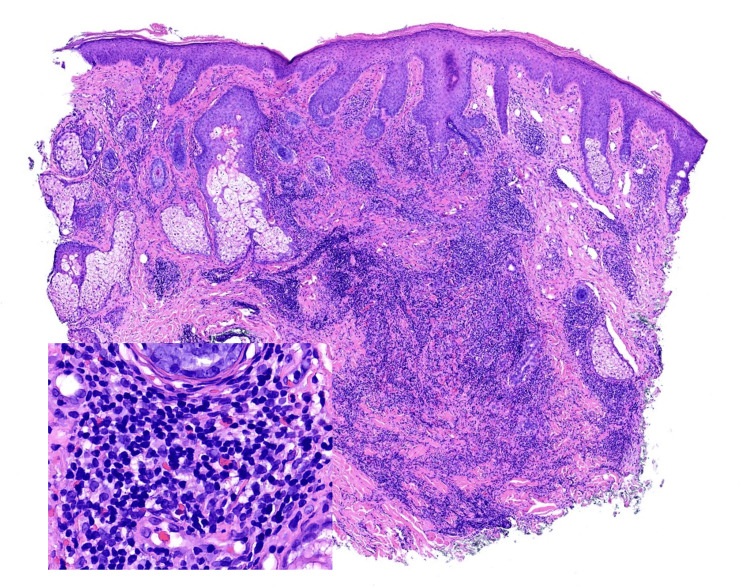
H&E ×1.5 and ×25 demonstrating a nodular infiltrate, composed of mature lymphocytes, histiocytes, plasma cells, and numerous eosinophils extending into the deep dermis. H&E: hematoxylin and eosin stain.

A 4 mm punch biopsy of the right cutaneous upper lip was initiated at the four-week follow-up. Pathology results depicted perivascular and periadnexal lymphocytic dermatitis with nodular mixed infiltrate in the mid-dermis containing lymphocytes, histiocytes, eosinophils, and plasma cells (Figure 2). An immunohistochemical workup demonstrated a mixed pattern, compatible with a reactive process. At the eight-week follow-up, 2+ inflammatory papules and nodules were distributed on the malar cheek bilaterally, along with centrofacial erythema and telangiectasias bilaterally distributed on the inferior central malar cheek. The differential diagnosis at this time included demodex folliculitis and rosacea, in which the patient began treatment with permethrin 5% topical cream daily and metronidazole 0.75% topical cream twice daily, while eosinophilic dermatosis of hematologic malignancy still remained high on the differential based on pathology and the patient’s underlying CLL. The patient’s otolaryngologist and oncologist were also notified of these results.

## Discussion

EDHM is an exceedingly rare chronic skin disorder affecting those with underlying hematologic malignancies, most notably chronic lymphocytic leukemia (CLL), as seen with our patient [5]. EDHM presents as a pruritic rash composed of macules, nodules, and sometimes vesicles or bullae. The location of affected areas varies and can include exposed and non-exposed areas on the body; however, the extremities are the most common site of outbreaks [3]. 

Initially, researchers hypothesized that EDHM was the result of CLL hypersensitivity to insect bites in the summer months [6]. This field of thought, however, began to change when many patients with these bullous, pruritic papules and vesicles reported no history of bites from insects. Thus, the term “insect-bite-like reaction” was adopted [7]. Byrd et al. further investigated this phenomenon and proposed criteria outlining EDHM: (A) pruritic papules, nodules, and/or vesiculobullous eruption refractory to standard treatment; (B) histopathology revealing eosinophil-rich superficial and deep dermal lymphohistiocytic infiltrate; (C) exclusion of other causes of tissue eosinophilia; and (D) diagnosis of hematologic malignancy [4,8].

Although the exact mechanism of EDHM is not well understood, it is commonly hypothesized that lymphocytic immune dysregulation is associated with recurrent episodes. TH2-associated cytokines such as IL-4, IL-5, and IL-13, which are often detectable in the presence of eosinophilia, may play a role in EDHM’s pathogenesis [9,10]. Increased IL-4 and IL-5 levels have also been linked to malignant B-cell and eosinophil proliferation, which could also explain the development of EDHM [3]. The pathology report from the lip punch biopsy performed on our patient returned findings consistent with perivascular and periadnexal lymphocytic dermatitis, with clusters of both CD3+ T cells and CD20+ B cells.

Treatment of this dermatosis can be difficult due to the natural relapsing nature of the hematologic malignancy associated with the reaction. There is also limited support from evidence-based medicine to guide management. Treatments used in reported cases of EDHM include antibiotics, immunoglobulin therapy, phototherapy, systemic and topical steroids, as well as interferon alpha [7].

## Conclusions

EDHM is a non-specific dermatosis characterized by erythematous vesicles, papules, or bullae. EDHM often presents alongside a hematologic disorder, most commonly CLL. Although the exact pathophysiology of EDHM is uncertain, it is thought that lymphocytic immune dysregulation plays an important role in its pathogenesis. The pathology report and clinical reports suggest that the patient has eosinophilic dermatosis, a hematologic malignancy associated with his underlying CLL. This often manifests as an exuberant skin reaction to various stimuli, such as bug bites or, in this patient’s case, likely rosacea or Demodex. The patient relates a history of intense redness and swelling following bug bites and corroborates this detail. We will focus on treating Demodex and rosacea with permethrin and metronidazole cream to see if he improves.

This case highlights the importance of considering EDHM in the differential diagnosis of persistent, pruritic skin lesions in patients with underlying hematologic malignancies. A multidisciplinary approach is essential for managing the condition. Treatment remains challenging, and reported approaches include the use of systemic and topical steroids, antibiotics, and immunomodulatory therapies.
